# Hedgehog in prostate cancer explained

**DOI:** 10.18632/oncoscience.405

**Published:** 2018-04-29

**Authors:** Ralph Buttyan, Na Li, Shabnam Massah

**Affiliations:** The Vancouver Prostate Centre, Vancouver BC V6H 3Z6 Canada

**Keywords:** prostate cancer, Hedgehog, Androgen Receptors, Gli, Steroid Receptors

Hedgehog (Hh) is an oncogenic cell signaling pathway causative of skin and brain cancers [1]. While long suspected to play a role in other solid tumors, including prostate cancer, the types of mutations in upstream Hh signaling elements, especially in *Patched* and *Smoothened (Smo)*, that drive hyperactive Hedgehog in skin and brain cancers are not found in these other tumors. When active, *Smo* suppresses a site- specific proteolysis of Gli2 and Gli3 that removes their C-terminal transactivation domains rendering them into functional repressors [2]. *Smo* action maintains Gli2/ Gli3 in high molecular weight, transcriptionally active forms that mediate the genomic effects of Hedgehog signaling. Recently, Li, et al [3] described a novel *Smo*- independent means of Gli activation in prostate cancer cells that is based upon the direct binding of androgen receptor (AR) proteins to Gli proteins. ARs (full-length liganded and constitutive-active truncated variants) recognize and bind to the C-terminal Protein Processing Domains (PPD) of Gli2 and Gli3. Furthermore it was shown that ARs effectively competed for Gli-PPD binding with the E3 ubiquitin ligase, β-TrCP, which marks Gli2/Gli3 for degradation. So AR binding to Gli2/ Gli3 effectively protects them from the constitutive degradation process that defines the Hedgehog inactive state and bypasses the need for upstream *Smo* activity. This identifies a new non-canonical means for Hedgehog activation in tumors and explains the failure of *Smo*- based therapeutics to affect progression of advanced prostate cancer [4]. More important, it shows that androgen action in prostate cancer is inexorably linked to the activity of Gli transcription factors.

The question remains as to whether the protection of Gli stability afforded by AR binding extends to the formation of transcription complexes on Gli-dependent genes. Preliminary ChIP studies have already shown that Gli-AR protein complexes are bound to Gli response elements (GliREs) on DNA of prostate cancer cells [5]. Here we need to determine whether the binding of Gli-AR complexes at GliREs is independent of the DNA binding activity of ARs or whether this interaction requires simultaneous DNA recognition and binding of both transcription factors. Our failure so far to identify concensus AREs proximal to concensus GliREs on Gli- dependent genes suggests that AR DNA binding may not be needed for activation of Gli transcription.

This finding recognizes a secondary function of the AR as a Gli co-activator in prostate cancer cells. It also raises interesting questions about other steroid- driven tumors, including breast cancer. Does the estrogen receptor (ER) share the Gli-activation potential of ARs? Furthermore, glucocorticoid receptor (GR) is thought to replace AR function in some hormone resistant prostate cancers [6]. Does GR provide the Gli-activation functions of AR in these instances? Resolution of these questions may explain reports of Hh activity in breast and other solid tumors and may ultimately impact on our understanding of the evolution of steroid receptors as AR is the evolutionary youngest of the steroid receptors whereas ER is the oldest.

We have yet to fully understand the biological function or impact of active Gli transcription in prostate cancer cells. Gli controls genes involved in embryonic development, apoptosis, cell motility and invasion and cell proliferation [7]. Each of these gene subsets play an important role in tumor progression and in the response of tumors to therapeutics. It is especially intriguing that Gli is an established regulator of cell growth. Indeed, almost all of the cyclin genes have been described as targets of Gli transcription as well as some cyclin- dependent kinases. This is likely the basis for Hh/Gli- dependent oncogenesis. So suppression of Gli may have significant benefit for patients with advanced and therapy-resistant prostate cancer.

Finally, the Li paper described a novel means of interference with the Gli-AR interaction that perturbed AR-driven activation of Gli in prostate cancer cells. Exogenous overexpression of a small AR-binding peptide from Gli2 competed with endogenous Gli3 for binding to AR and suppressed activation of a Hh/Gli reporter and expression of Gli-dependent genes. This suggests a possible therapeutic approach to diminish Gli activity in prostate cancer cells. While the peptide approach is an effective proof-of-principle, discovery of a viable small molecule approach will likely be more practical for clinical application to repress Gli activity and its effects in patients with prostate cancer. Such a narrow therapeutic approach might avoid the complications of systemic Hh/Gli suppression already identified in oncology patients treated with *Smo* inhibitors.
